# CloudProteoAnalyzer: scalable processing of big data from proteomics using cloud computing

**DOI:** 10.1093/bioadv/vbae024

**Published:** 2024-02-23

**Authors:** Jiancheng Li, Yi Xiong, Shichao Feng, Chongle Pan, Xuan Guo

**Affiliations:** Department of Computer Science and Engineering, University of North Texas, Denton, TX 76203, United States; School of Biological Sciences, University of Oklahoma, Norman, OK 73019, United States; Department of Computer Science and Engineering, University of North Texas, Denton, TX 76203, United States; School of Biological Sciences, University of Oklahoma, Norman, OK 73019, United States; School of Computer Science, University of Oklahoma, Norman, OK 73019, United States; Department of Computer Science and Engineering, University of North Texas, Denton, TX 76203, United States

## Abstract

**Summary:**

Shotgun proteomics is widely used in many system biology studies to determine the global protein expression profiles of tissues, cultures, and microbiomes. Many non-distributed computer algorithms have been developed for users to process proteomics data on their local computers. However, the amount of data acquired in a typical proteomics study has grown rapidly in recent years, owing to the increasing throughput of mass spectrometry and the expanding scale of study designs. This presents a big data challenge for researchers to process proteomics data in a timely manner. To overcome this challenge, we developed a cloud-based parallel computing application to offer end-to-end proteomics data analysis software as a service (SaaS). A web interface was provided to users to upload mass spectrometry-based proteomics data, configure parameters, submit jobs, and monitor job status. The data processing was distributed across multiple nodes in a supercomputer to achieve scalability for large datasets. Our study demonstrated SaaS for proteomics as a viable solution for the community to scale up the data processing using cloud computing.

**Availability and implementation:**

This application is available online at https://sipros.oscer.ou.edu/ or https://sipros.unt.edu for free use. The source code is available at https://github.com/Biocomputing-Research-Group/CloudProteoAnalyzer under the GPL version 3.0 license.

## 1 Introduction

Shotgun proteomics enables comprehensive identification and quantification of numerous proteins in biological samples, providing valuable insights into cellular processes and metabolism regulations (Aebersold and Mann 2016, Zelezniak *et al.* 2018). Experimentally, the proteins extracted from a laboratory culture or an environmental sample are digested into a complex peptide mixture by proteases. Peptides are then separated using liquid chromatography and analyzed by a mass spectrometer. Computationally, the acquired tandem mass spectral data (MS/MS) is then searched against a protein database to identify the peptides in the peptide mixture. These identified peptides are then assembled into proteins in the metaproteome of the original biological sample. The relative abundances of the identified proteins can be estimated across samples from multiple biological conditions. Many algorithms, such as Comet (Eng *et al.* 2013), MSFragger (Kong *et al.* 2017), MS-GF+ (Kim and Pevzner 2014), pFind3 (Chi *et al.* 2018), and Sipros Ensemble (Guo *et al.* 2018), have been devised for protein identification, while others like ProRata (Pan *et al.* 2006), IonQuant (Yu *et al.* 2021), and MaxQuant (Tyanova *et al.* 2016) assist in protein quantification. pFind3 can support both open and restricted search modes for database searching, along with multiple types of quantification. FragPipe, a pipeline designed for identification and quantification, can combine various post-processing methods and different types of quantification. However, most of the existing proteomics algorithms are designed for manual installation and execution on a local computer. The limited computing capability constrains the scalability and throughput of proteomics data processing. In addition, the intricate toolchain spanning from the raw data to the final results is a challenge for biologists in both installation and operation aspects. To overcome these challenges, scientists have developed several cloud-based proteomics tools, such as SQuAPP (Ergin *et al.* 2022), Bioconda (Grüning *et al.* 2018) with Biocontainers (da Veiga Leprevost *et al.* 2017), and MaxQuant integrated with Galaxy (Afgan *et al.* 2018). Bioconda constitutes a Conda software package expressly crafted for the analysis of proteomics data utilizing the TPP toolset’s command-line versions. It is compatible with biocontainers and deployable across individual machines as well as cloud-based platforms. SQuAPP is a web application that can analyze protein quantification results derived from alternative tools. MaxQuant has been adapted to Galaxy, a web-based platform for bioinformatics. Nonetheless, Galaxy-based MaxQuant has limited support for large protein databases commonly encountered in metaproteomics.

Our platform, called CloudProteoAnalyzer, was developed to provide a convenient online service for proteomics data analysis. The backend of CloudProteoAnalyzer was implemented within a high-performance computing (HPC) cluster to enhance computational scalability for database searching. Database searching was distributed across multiple computing nodes and multi-threaded on multi-core CPUs. The user-friendly web interface of CloudProteoAnalyzer empowers users to customize all the parameters for protein identification and quantification.

## 2 Software framework and implementation

**Figure 1.**
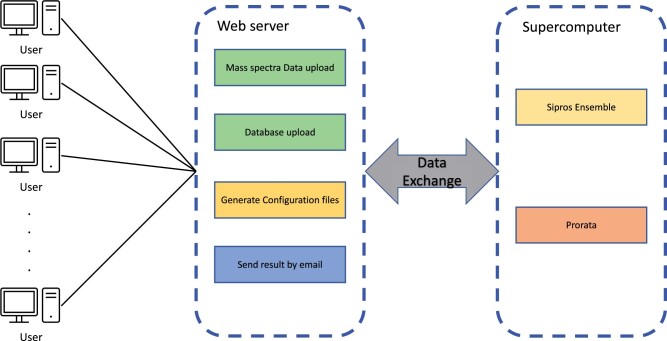
CloudProteoAnalyzer architecture. Two main components: the web server for supporting users to log in and manage data, and the supercomputer for supporting identification and quantification processes.

CloudProteoAnalyzer was developed using Python, the Streamlit library, and Apache Tomcat to provide a user-friendly web application running on the cloud and the supercomputer. CloudProteoAnalyzer comprises two main components, as illustrated in Fig. 1. The first component functions as the web server, which provides a graphical user interface in a browser and facilitates data transfer between users and the supercomputer. The other component is tasked with allocating computational resources and executing Sipros Ensemble and ProRata on the supercomputer. The functionalities of the web server encompass user login, storage of user information, generation of configuration files for identification and quantification, data transfer with the supercomputer, and results delivery via email. CloudProteoAnalyzer executes protein identification and quantification in the following steps. First, it automatically converts Thermo Fisher Scientific raw files to suitable inputs for Sipros Ensemble and ProRata. Second, it constructs new protein databases containing decoys and target peptides. Third, it uses user input to generate configuration files for executing Sipros Ensemble and ProRata, as well as Slurm script files for operating these two programs on the supercomputer. The responsibility of executing all identification and quantification tasks on single or multiple computing nodes using OpenMP and MPI lies with a supercomputer. Slurm, an open-source task scheduler commonly employed on supercomputers, schedules and executes all identification and quantification tasks. During MPI execution, the number of tasks equals the total number of MS2 files multiplied by the number of protein database files. These tasks are then evenly distributed among the computing nodes. One MS2 file searches against one protein database file in each task. Multiple tasks can be parallelly executed on each computing node. The results generated by one task are written into one file. All database search results are gathered from all computing nodes and are combined into one file by a Python script.

Users are required to log in using their Google accounts. Prior to login, users need to upload the raw MS/MS files and protein databases without decoy proteins to their Google Drive. Following successful login, users can customize the parameters associated with protein identification and quantification through a web interface. Users have the option to choose whether to execute the program on one or multiple computing nodes of the supercomputer. In addition, users can opt for various protein identification and quantification settings, such as post-translational modification (PTM) identification and label-free quantification. Upon clicking the “run” button, CloudProteoAnalyzer automatically transfers all converted mass spectral data, protein databases, and configuration files from the web server to the supercomputer for execution. Users can then close their web browsers and wait for the results. CloudProteoAnalyzer monitors the running status of Sipros Ensemble and ProRata. Upon the completion of the analysis, CloudProteoAnalyzer dispatches tabulated files to users via email. These files encapsulate the outcomes of peptide and protein identification and quantification.

## 3 Experimental and result

A comparative analysis was conducted between CloudProteoAnalyzer and three proteomics tools: MaxQuant on Galaxy cluster (denoted as M/G in this article), pFind3, and FragPipe across four datasets including the UPS1 spiked in yeast dataset (Pursiheimo *et al.* 2015), the MaxQuant label-free dataset (Tyanova *et al.* 2016), a soil metaproteome dataset, and a marine metaproteome dataset (Guo *et al.* 2018). The UPS1 spiked-in yeast dataset was used for comparing label-free quantification results. Supplementary information regarding the datasets can be found in the Supplementary Note. FragPipe was run on a single node of a supercomputer which is the same as CloudProteoAnalyzer run. The pFind3 was executed on a Windows 11 system with an 8-Core 4.0 GHz CPU, 32GB 3200 MHz RAM, and NVMe 3.0 SSD due to it only running on Windows.

**Figure 2.**
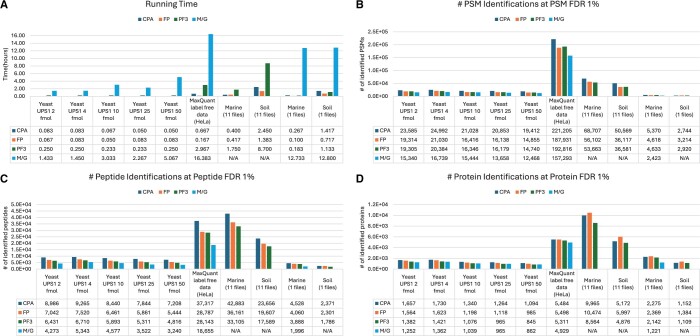
Running time and performance comparison. CloudProteoAnalyzer is denoted as CPA. FragPipe is denoted as FP. pFind3 is denoted as PF3. MaxQuant on Galaxy is denoted as M/G.

The running time, as shown in Fig. 2, was compared between the four methods. We executed the MPI version of CloudProteoAnalyzer on two computing nodes. CloudProteoAnalyzer was significantly faster than M/G because CloudProteoAnalyzer used two computer nodes with a total of 40 cores. MaxQuant’s computational velocity is constrained due to its lack of MPI. Due to the 24-hour restriction inherent in Galaxy, M/G encountered failure in analyzing 11 raw files from the soil dataset and 11 raw files from the marine dataset. In total, CloudProteoAnalyzer is faster than pFind3. The pFind3 can only run on a local Windows machine. Although FragPipe runs faster than CloudProteoAnalyzer, FragPipe does not run on distributed computer cloud due to its lack of support for MPI. CloudProteoAnalyzer, on the other hand, can achieve faster performance by scaling up the number of computer nodes. The computational horizontal scaling is shown in Supplementary Fig. S3. The scalability of CloudProteoAnalyzer was tested on the HPC system using the soil dataset. CloudProteoAnalyzer demonstrated nearly linear speed-up in its computations using up to 12 compute nodes and 240 CPU cores.

We conducted a comprehensive analysis of PSMs, peptides, and proteins identified by four methods at 1% FDR, with the results illustrated in Fig. 2. Regarding PSM, peptide, and protein identification, we used the false discovery rate control and the protein assembly procedure in CloudProteoAnalyzer for FragPipe and pFind3 to ensure a fair comparison. The outputs of M/G are filtered, so we can not use FDR control from CloudProteoAnalyzer. The findings revealed that CloudProteoAnalyzer consistently outperformed the other three methods across various datasets. On average, it identified 21% more PSMs and 23% more peptides than the second-best method. CloudProteoAnalyzer utilizes three scoring functions (Guo *et al.* 2018)—the cross-correlation score from Comet, the multivariate hypergeometric score from MyriMatch, and the weighted dot product score from the original Sipros. In contrast, each of the other methods relied on a single score: the hyperscore from MSFragger in FragPipe, the EV-Score in pFind3, and the Andromeda score in MaxQuant. The multi-score approach of CloudProteoAnalyzer contributed to increased PSM and peptide identifications. A protein is considered identified if at least one unique peptide from that protein is identified. As shown in Fig. 2, CloudProteoAnalyzer consistently outperformed or was comparable to other methods in protein identification, demonstrating an average increase of 0.8% over the second-best method. M/G identified the lowest numbers of PSMs, peptides, and proteins because tolerance, the important parameter for identification, cannot be changed. To further investigate the peptide identifications reported only by CloudProteoAnalyzer, we calculated the ratio of the peptide count per identified protein between CloudProteoAnalyzer and the second-best method. We found that this ratio was very close to the ratio of the number of identified peptides between CloudProteoAnalyzer and the second-best method. For example, the ratio of the peptide count per identified protein was 1.25, and the ratio of the number of identified peptides was 1.29 on the MaxQuant label-free dataset. In brief, CloudProteoAnalyzer identified more peptides per protein, leading to improved protein quantification.

As a supplement to the target-decoy strategy, we conducted the entrapment database search. All methods produced similar results. The details about the entrapment database search and the results are shown in Supplementary Note 3 and Supplementary Tables S1–S3. In brief, all benchmarked methods effectively controlled the false match rate.

Finally, we conducted a comparison of quantification results using UPS1 spiked in the yeast dataset based on the ratio of the protein abundances across three replicates, as shown in Supplementary Figs S4–S13. We compared the results from CloudProteoAnalyzer, FragPipe, and M/G as pFind3 only performs quantification at the peptide level. CloudProteoAnalyzer and M/G employed default quantification parameters, while FragPipe used LFQ-MBR workflow for quantification. In general, the standard deviation of the intensity ratios of M/G was slightly better than that of CloudProteoAnalyzer and FragPipe across all comparisons. The averaged intensity ratios of the three methods were very close for all comparisons. Furthermore, accuracy was assessed by comparing the differences between the median of the observed protein ratios and the theoretical protein ratios, and precision was evaluated based on the standard deviation of the median of the observed protein ratios. All results are shown in Supplementary Table S4. The last line of the table showed the average of all the experiments. Although M/G achieved better accuracy and precision, the results of CloudProteoAnalyzer closely approached the results of the M/G. Overall, the quantification results of CloudProteoAnalyzer were comparable to those of M/G and FragPipe.

## 4 Discussion

The security of data is crucial for cloud-based applications. To protect data privacy, CloudProteoAnalyzer utilizes Google Drive and email to transmit data and results. Furthermore, all data and results are promptly deleted from the server and supercomputer after being sent out.

CloudProteoAnalyzer can analyze Thermo Fisher mass spectrometry data. Currently, it exclusively analyzes data-dependent acquisition MS/MS data and supports several well-known PTM, such as Oxidation of Met, Deamidation of NQ, Mono-methylation, and so on. In the future, we will add functions to accept more types of MS/MS data from different mass spectrometers, such as AB Sciex, and various file formats like mzML and mzXML. So far, our platform supports protein identification and quantification but does not include downstream statistical analysis. We will extend our platform with a statistical analysis function, presenting results through tables and figures.

## 5 Conclusion

CloudProteoAnalyzer is a cloud platform that provides a user interface and precise analysis of large-scale proteomics data. Within CloudProteoAnalyzer, users can upload proteomics data, configure parameters, submit jobs, and monitor job status. The data processing is distributed across multiple computing nodes in a supercomputer to achieve scalability for extensive datasets. The experimental findings demonstrated that software as a service for proteomics is a feasible solution for the community seeking to scale up data processing.

## Supplementary Material

vbae024_Supplementary_Data

## Data Availability

All data are described in supplementary data.
