# Supplementary material for: CloudProteoAnalyzer: scalable processing of big data from proteomics using cloud computing
Source: Bioinform Adv. 2024 Feb 23;4(1):vbae024. doi: 10.1093/bioadv/vbae024 (PMC10942798; doi:10.1093/bioadv/vbae024)
Supplement: vbae024_Supplementary_Data [file vbae024_supplementary_data.pdf]

CloudProteoAnalyzer: scalable processing of big data from proteomics using cloud computing

February 19, 2024

Datasets

Yeast and UPS1 dataset

The dataset was downloaded from PRIDE data repository (ID: PXD002099). All mass spectrometry data were used to evaluate the results between CloudProteoAnalyzer, FragPipe, pFind3, and MaxQuant on Galaxy.

MaxQuant label-free dataset

The dataset was downloaded from PRIDE data repository (ID: PXD000612). We used mass spectrometry data from 20111219_EXQ5_KiSh_SA_LabelFree_HeLa_Proteome_Control_rep1_pH3.raw to 20120125_EXQ5_KiSh_SA_LabelFree_HeLa_Proteome_Control_rep6_pH3.raw with same PH value to evaluate results between CloudProteoAnalyzer, FragPipe, pFind3, and MaxQuant on Galaxy.

Soil dataset

The dataset was downloaded from PRIDE data repository (ID: PXD002099) to evaluate the results between CloudProteoAnalyzer, FragPipe, pFind3, and MaxQuant on Galaxy. The evaluation was carried out using all the available mass spectrometry data, including 11 runs from Angelo_08202013_P1_3040cm_MB_FASP_Elite_Run1_01.raw to Angelo_08202013_P1_3040cm_MB_FASP_Elite_Run1_11.raw. In addition, we used 1 mass spectrometry data, Angelo_08202013_P1_3040cm_MB_FASP_Elite_Run1_05.raw to evaluate results.

Marine dataset

The dataset was downloaded from PRIDE data repository (ID: PXD002099) and was used to evaluate the performance between CloudProteoAnalyzer, FragPipe, pFind3, and MaxQuant on Galaxy. All the mass spectrometry data available was used, including 11 runs from OSU_D7_FASP_Elite_03172014_01.raw to OSU_D7_FASP_Elite_03172014_11.raw. In addition, we used 1 mass spectrometry data, OSU_D7_FASP_Elite_03172014_06.raw to evaluate results.

Supplementary Note 1

All configuration files, protein databases, and script files can be downloaded from our GitHub at https://github.com/Biocomputing-Research-Group/CloudProteoAnalyzer.

Identification and quantification parameters

The parameters for identification and quantification are detailed in this section. For the Yeast and UPS1 dataset, the four methods were executed with the following parameters: only tryptic peptides allowed, up to 2 missed cleavages, one oxidation of methionine as variable modifications, setting 0.05 Da precursor tolerance, setting 0.5 Da fragment tolerance, and peptide sequences ranging from 7 to 65. Each protein is assembled with 1 peptide. For the MaxQuant label-free dataset, the four methods were executed with the following parameters: only tryptic/p peptides allowed, up to 1 missed cleavage, one oxidation of methionine as variable modifications, setting 0.05 Da precursor tolerance, setting 0.01 Da fragment tolerance, and peptide sequences ranging from 7 to 58. Each protein is assembled with 1 unique peptide. For the marine and soil datasets, the four methods were executed with the following parameters: only tryptic/p peptides allowed, up to 3 missed cleavages, one oxidation of methionine as variable modifications, setting 0.09 Da precursor tolerance, setting 0.01 Da fragment tolerance, and peptide sequences ranging from 7 to 60. Each protein is assembled with 1 unique peptide.

Identification workflow

We used raw mass spectrometry data and a protein database without decoy sequences to execute the CloudProteoAnalyzer.

We executed the built-in default workflow in FragPipe (version v20.0) with a protein database including decoy sequences and mass spectrometry data in mzML format. The decoy sequences were reversed from the original protein database. Raw data was converted to mzML format using the ProteoWizard package (version 3.0.20323). Outputs from Percolator were utilized as input for "fragpipe2sipros.py" and "sipros_peptides_assembling.py" scripts to control FDR at PSM, peptide, and protein levels.

We used raw mass spectrometry data and a protein database without decoy sequences to execute pFind (version 3.2.0) without an open search. Subsequently, we exported PSM score files from pFind as input for "pfind2sipros.py" and "sipros_peptides_assembling.py" scripts to control FDR at PSM, peptide, and protein levels.

We used the raw mass spectrometry data and a protein database without decoy sequences to execute MaxQuant on Galaxy (https://usegalaxy.org/).

Quantification workflow

For CloudProteoAnalyzer, we executed quantitative analyses following the identification workflow described above. The default parameters of quantification are used.

We executed the built-in LFQ-MBR workflow in FragPipe (version v20.0) with a protein database with decoy sequences and mass spectrometry data in mzML format.

For PFind3, we executed quantitative analyses following the identification workflow described above. The quantification type was set to label-free, and "TYPE_SAME_START_END_BETWEEN_EVIDENCE" was set to independent.

We executed label-free quantitative analyses following the identification workflow described above in MaxQuant on Galaxy. The default parameters of label-free quantification are used.

Supplementary Note 2

The CloudProteoAnalyzer calculates three scores and seven additional features for each PSM during database searching and filtering. The scores and additional features are listed below:

- $Xcorr$: cross-correlation score from Comet;
- MVH: multivariate hypergeometric score from MyriMatch;
- WDP: weighted dot product score from the original Sipros;
- ΔMVH: score differential for the MVH score by Equation 1 below;
- $\Delta Xcorr$: score differential for the Xcorr score by Equation 1 below;
- ΔWDP: score differential for the WDP score by Equation 1 below;
- ΔM: the absolute difference between the calculated and observed masses of the precursor ion;
- $\#MCS$: number of missed cleavage sites in the peptide of the PSM;
- $\#PEP$: spectrum count for the peptide of the PSM, including all modification forms and charge states of the peptide;
- $\#PRO$: spectrum counts for the protein or the protein group of the PSM. The spectrum count of a protein or a protein group includes both unique peptides and shared peptides. If this PSM can be assigned to multiple proteins or protein groups, the one with the highest spectrum count is used.

The score differential of a PSM for a given scoring function is calculated as:

$$\Delta = \frac{S_p - S_h}{S_h} \tag{1}$$

where S_p is the score of the PSM and S_h is the highest score of other PSMs for this spectrum.

For the PSM filtering, a logistic binary classifier with L2 regularization is trained using the above features. The positive dataset consists of the PSMs top-ranked by all three scoring functions. The negative dataset consists of the decoy PSMs with the reversed proteins. The trained classifier is used to evaluate the top-ranking PSM(s) from the three scoring functions for every spectrum in a

proteomics run. For a spectrum with two or three top-ranking PSMs identified by different scoring functions, the PSM with the highest classification score is selected as the top-ranking PSM for filtering. Every spectrum has one and only one PSM for filtering based on its classification score. The score threshold is adjusted to reach a user-defined FDR. The FDR of a set of filtered PSM is calculated as

$$FDR = \frac{\alpha \times \#TestDecoy}{\#Target} \quad (2)$$

where $\#Target$ is the number of target PSMs, $\#TestDecoy$ is the number of decoy PSMs in the testing dataset. In our experiments, α was set to 2 because the entire set of decoy proteins was evenly split into the training and testing datasets. A peptide is identified if any of its PSMs is identified. A protein is identified if at least one unique peptide from this protein is identified.

Supplementary Note 3

In the entrapment search, the target database was bipartite, comprising a sample partition (containing known protein sequences) and an entrapment partition (consisting of a repeatedly shuffled sample partition). The decoy database was constructed by reversing both the sample and entrapment partitions. Top-scoring PSMs matching the sample sequences were considered correct, while those matching the entrapment sequences were deemed incorrect. The false match rate (FMR) was calculated as the number of entrapments divided by the total of target matches at a 1% FDR. The results were presented in Tables S1–S3. All four methods demonstrated comparable precision with FMR lower than the user-defined FDR.

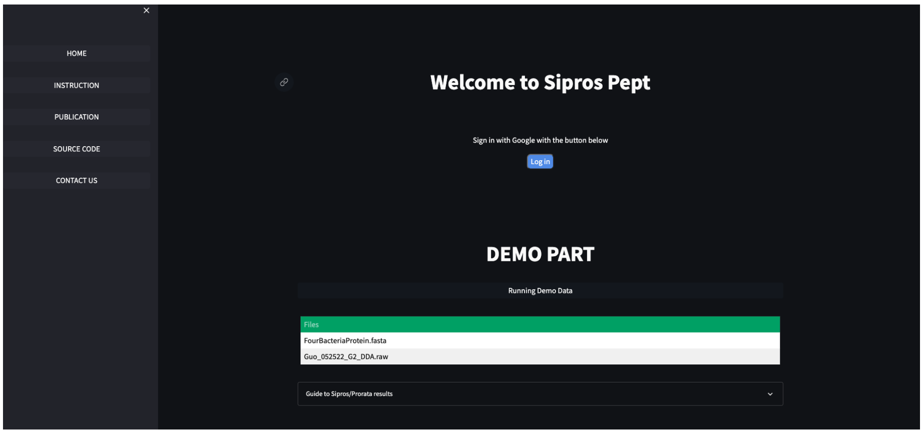

Figure S1: The login Page.

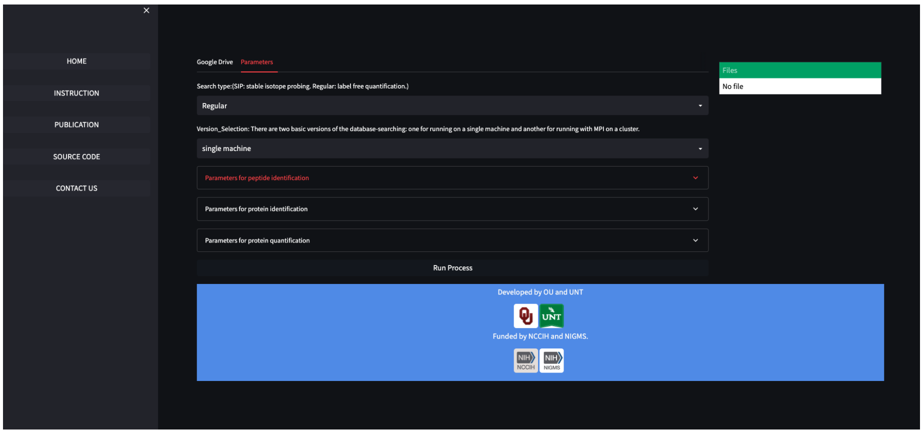

Figure S2: The parameter setting page.

Table S1: False Match Rate of PSMs

	CPA	FP	PF3	M/G
Yeast UPS1 2 fmol	0.0076	0.0076	0.0082	0.0083
Yeast UPS1 4 fmol	0.0067	0.0094	0.0093	0.0052
Yeast UPS1 10 fmol	0.0102	0.0083	0.0088	0.0052
Yeast UPS1 25 fmol	0.0100	0.0104	0.0120	0.0092
Yeast UPS1 50 fmol	0.0105	0.0076	0.0123	0.0104
MaxQuant label-free data (HeLa)	0.0074	0.0075	0.0086	0.0016

CloudProteoAnalyzer is denoted as CPA.
FragPipe is denoted as FP.
pFind3 is denoted as PF3.
MaxQuant on Galaxy is denoted as M/G.

Table S2: False Match Rate of Peptides

	CPA	FP	PF3	M/G
Yeast UPS1 2 fmol	0.0075	0.0071	0.0092	0.0101
Yeast UPS1 4 fmol	0.0076	0.0102	0.0127	0.0079
Yeast UPS1 10 fmol	0.0092	0.0073	0.0082	0.0076
Yeast UPS1 25 fmol	0.0115	0.0129	0.0111	0.0131
Yeast UPS1 50 fmol	0.0096	0.0070	0.0103	0.0133
MaxQuant label-free data (HeLa)	0.0022	0.0023	0.0081	0.0025

CloudProteoAnalyzer is denoted as CPA.
FragPipe is denoted as FP.
pFind3 is denoted as PF3.
MaxQuant on Galaxy is denoted as M/G.

Table S3: False Match Rate of Proteins

	CPA	FP	PF3	M/G
Yeast UPS1 2 fmol	0.0103	0.0074	0.0234	0.0355
Yeast UPS1 4 fmol	0.0069	0.0108	0.0290	0.0272
Yeast UPS1 10 fmol	0.0120	0.0095	0.0243	0.0310
Yeast UPS1 25 fmol	0.0161	0.0147	0.0302	0.0483
Yeast UPS1 50 fmol	0.0094	0.0128	0.0309	0.0511
MaxQuant label-free data (HeLa)	0.0063	0.0068	0.0054	0.0080

CloudProteoAnalyzer is denoted as CPA.
FragPipe is denoted as FP.
pFind3 is denoted as PF3.
MaxQuant on Galaxy is denoted as M/G.

Table S4: Accuracy and Precision of Quantification

	Yeast protein ratio accuracy			Yeast protein ratio precision			UPS1 protein ratio accuracy			UPS1 protein ratio precision		
	CPA	M/G	FP	CPA	M/G	FP	CPA	M/G	FP	CPA	M/G	FP
Yeast UPS1 2 fmol - 4 fmol	0.35	0.35	0.37	0.92	0.45	0.66	0.19	0.11	0.36	0.54	0.31	0.64
Yeast UPS1 2 fmol - 10 fmol	1.02	1.14	1.04	1.2	0.68	0.95	0.3	0.46	0.12	0.7	0.45	0.93
Yeast UPS1 2 fmol - 25 fmol	1.55	1.24	1.28	1.25	0.7	0.99	0.27	0.02	0.01	0.78	0.54	0.77
Yeast UPS1 2 fmol - 50 fmol	1.92	1.53	1.78	1.34	0.74	0.96	0.72	0.55	0.76	0.81	0.52	0.79
Yeast UPS1 4 fmol - 10 fmol	1.34	1.5	1.48	1.16	0.67	0.99	0.38	0.49	0.78	0.66	0.51	1.13
Yeast UPS1 4 fmol - 25 fmol	1.89	1.6	1.69	1.19	0.61	1.02	0.57	0.09	0.41	0.69	0.52	0.92
Yeast UPS1 4 fmol - 50 fmol	2.26	1.76	2.16	1.26	0.62	1.01	1.04	0.55	1.03	0.67	0.61	1.25
Yeast UPS1 10 fmol - 25 fmol	0.51	0.12	0.25	1.05	0.55	0.82	0.02	0.44	0.11	0.53	0.44	0.75
Yeast UPS1 10 fmol - 50 fmol	0.93	0.34	0.72	1.19	0.64	0.83	0.49	0.04	0.52	0.5	0.053	0.88
Yeast UPS1 25 fmol - 50 fmol	0.3	0.21	0.48	0.93	0.44	0.59	0.47	0.42	0.72	0.29	0.25	0.6
Average	1.21	0.98	1.13	1.15	0.61	0.88	0.45	0.32	0.48	0.62	0.42	0.87

CloudProteoAnalyzer is denoted as CPA.
FragPipe is denoted as FP.
pFind3 is denoted as PF3.
MaxQuant on Galaxy is denoted as M/G.

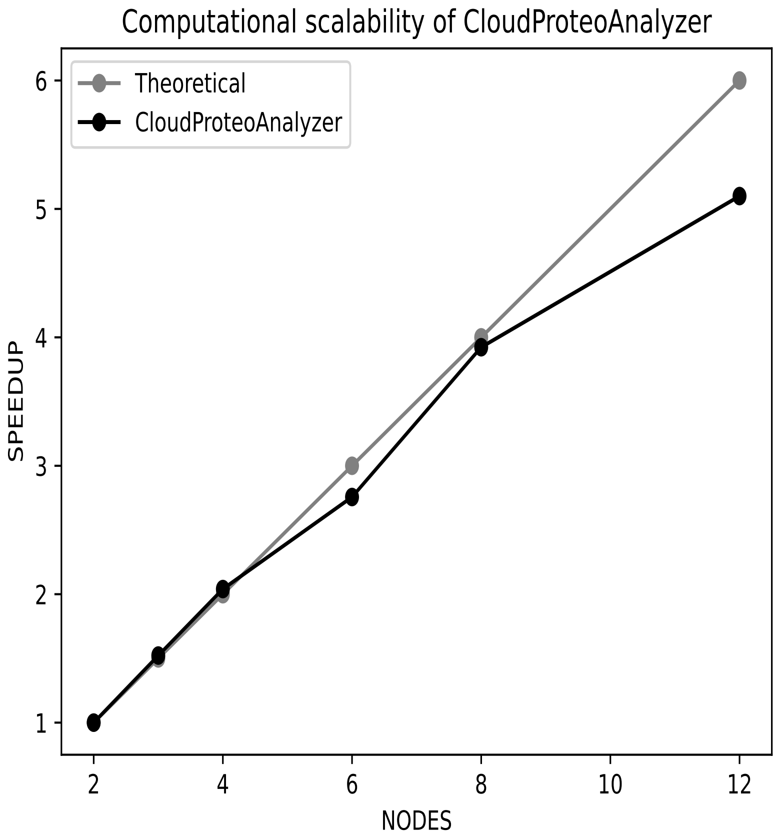

Figure S3: Computational scalability of CloudProteoAnalyzer on a supercomputer

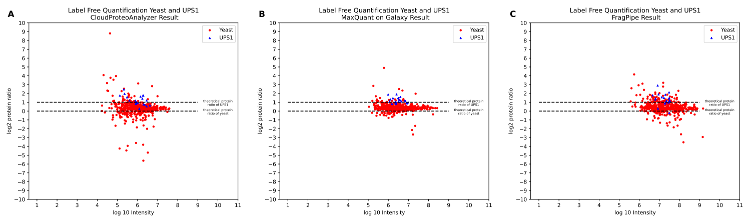

Figure S4: Scatter plots of log-transformed ratio between $2fmol/\mu L$ and $4fmol/\mu L$ UPS1 proteins spiked in yeast proteins. The theoretical protein ratio of the yeast is 0, and the theoretical protein ratio of the UPS1 is 1.

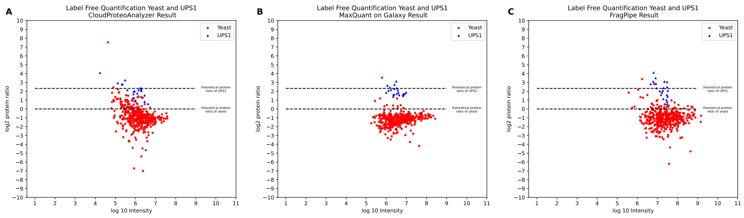

Figure S5: Scatter plots of log-transformed ratio between $2fmol/\mu L$ and $10fmol/\mu L$ UPS1 proteins spiked in yeast proteins.The theoretical protein ratio of the yeast is 0, and the theoretical protein ratio of the UPS1 is 2.32.

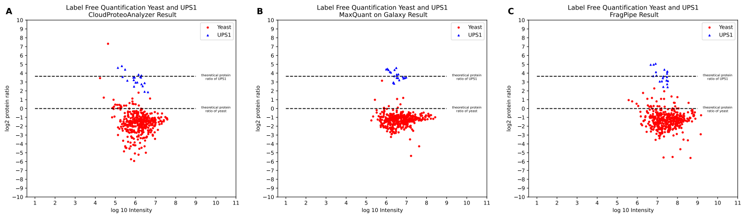

Figure S6: Scatter plots of log-transformed ratio between $2fmol/\mu L$ and $25fmol/\mu L$ UPS1 proteins spiked in yeast proteins.The theoretical protein ratio of the yeast is 0, and the theoretical protein ratio of the UPS1 is 3.64.

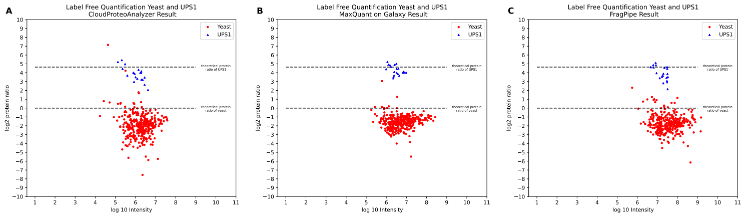

Figure S7: Scatter plots of log-transformed ratio between $2fmol/\mu L$ and $50fmol/\mu L$ UPS1 proteins spiked in yeast proteins.The theoretical protein ratio of the yeast is 0, and the theoretical protein ratio of the UPS1 is 4.64.

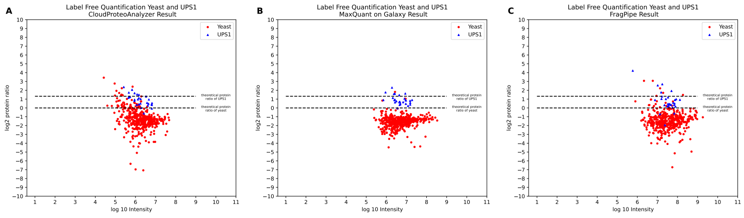

Figure S8: Scatter plots of log-transformed ratio between $4fmol/\mu L$ and $10fmol/\mu L$ UPS1 proteins spiked in yeast proteins.The theoretical protein ratio of the yeast is 0, and the theoretical protein ratio of the UPS1 is 1.32.

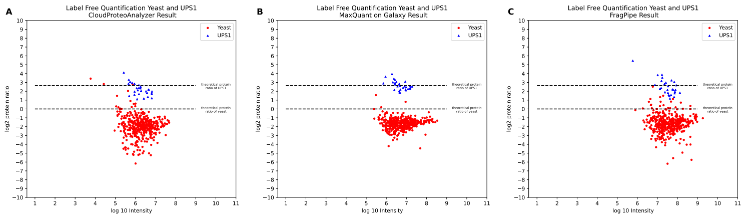

Figure S9: Scatter plots of log-transformed ratio between $4fmol/\mu L$ and $25fmol/\mu L$ UPS1 proteins spiked in yeast proteins.The theoretical protein ratio of the yeast is 0, and the theoretical protein ratio of the UPS1 is 2.64.

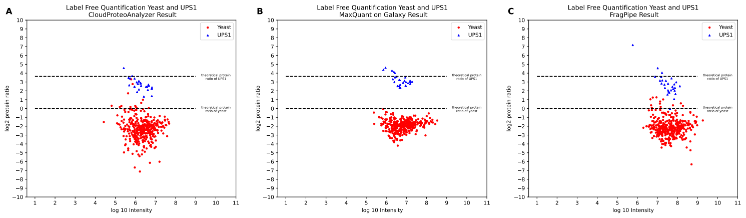

Figure S10: Scatter plots of log-transformed ratio between $4fmol/\mu L$ and $50fmol/\mu L$ UPS1 proteins spiked in yeast proteins.The theoretical protein ratio of the yeast is 0, and the theoretical protein ratio of the UPS1 is 3.64.

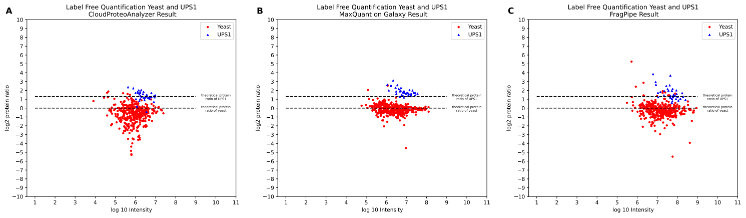

Figure S11: Scatter plots of log-transformed ratio between $10fmol/\mu L$ and $25fmol/\mu L$ UPS1 proteins spiked in yeast proteins.The theoretical protein ratio of the yeast is 0, and the theoretical protein ratio of the UPS1 is 1.32.

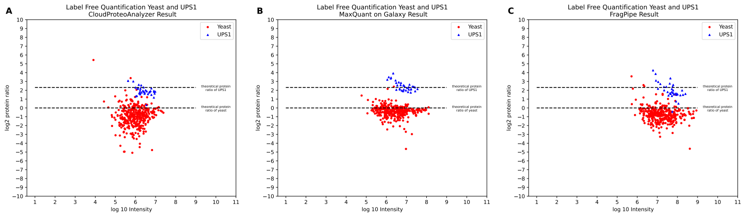

Figure S12: Scatter plots of log-transformed ratio between $10fmol/\mu L$ and $50fmol/\mu L$ UPS1 proteins spiked in yeast proteins.The theoretical protein ratio of the yeast is 0, and the theoretical protein ratio of the UPS1 is 2.32.

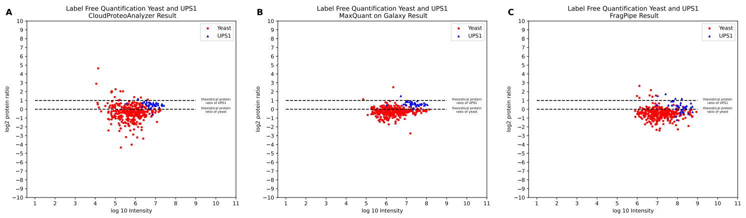

Figure S13: Scatter plots of log-transformed ratio between $25fmol/\mu L$ and $50fmol/\mu L$ UPS1 proteins spiked in yeast proteins.The theoretical protein ratio of the yeast is 0, and the theoretical protein ratio of the UPS1 is 1.
